# Responses of signal crayfish *Pacifastacus leniusculus* to single short-term pulse exposure of pesticides at environmentally relevant concentrations

**DOI:** 10.1007/s11356-023-25908-7

**Published:** 2023-02-23

**Authors:** Viktoriia Malinovska, Iryna Kuklina, Filip Lozek, Josef Velisek, Pavel Kozak

**Affiliations:** Faculty of Fisheries and Protection of Waters, South Bohemian Research Center of Aquaculture and Biodiversity of Hydrocenoses, University of South Bohemia in Ceske Budejovice, Zatisi 728/II, 389 25 Vodnany, Czech Republic

**Keywords:** Freshwater invertebrate, Locomotor activity Metazachlor, Short-term exposure, Terbuthylazine, Thiacloprid

## Abstract

Although pesticides are often discharged into surface waters in pulses as opposed to a sustained release, the effect of episodic pollution events on freshwater crayfish is largely unknown. We monitored change in heart rate and distance moved to assess the response of signal crayfish *Pacifastacus leniusculus* to short-term exposure to environmentally relevant concentrations of metazachlor (MTZ), terbuthylazine (TER), and thiacloprid (TCL). Crayfish exposed to 20 µg/L of MTZ exhibited a significant increase in mean heart rate and distance moved. Increased heart rate was detected at 118 ± 74 s post-exposure to MTZ. There were no significant differences in mean heart rate and distance moved in crayfish exposed to 6 µg/L of TCL and 4 µg/L of TER. A significant correlation between heart rate and distance moved was found in all exposed groups. These results suggest that pulse exposure to MTZ impact crayfish physiology and behavior during short-term period. With pulse exposure to TCL and TER, crayfish not exhibiting a locomotor response may continue to be exposed to lower, but potentially harmful, levels of pollutants. Evidence of the impacts of pesticide pulse at environmentally relevant concentrations on crayfish is scarce. Further study is required to determine the ecological effects of such events on freshwater crayfish.

**Supplementary Information:**

The online version contains supplementary material available at 10.1007/s11356-023-25908-7.

## Introduction

Runoff of contaminants from agricultural land into aquatic ecosystems has long been a concern (Gao et al. 2008; Matin et al. 1998; Palma et al. 2014; Wan et al. 2021), and the impact has accelerated with the expansion of cultivated areas and accompanying increase in the application of agrochemicals (Benbrook 2016; Dobrovolski et al. 2001; Oerke 2006). Numerous studies provide evidence of pesticide residues in surface waters worldwide (De Geronimo et al. 2014; Herrero-Hernandez et al. 2020; Jergentz et al. 2005; Papadakis et al. 2018) with concentrations often exceeding the safety levels (Jergentz et al. 2005; Papadakis et al. 2018). Agricultural activities have been shown to induce significant adverse effects on non-target species, including crayfish (Bunzel et al. 2015; Rosi-Marshall et al. 2007; Sohn et al. 2018; Stara et al. 2019). Exposure to pesticides can result in behavioral, biochemical, and histological alterations in freshwater crayfish (Sohn et al. 2018; Stara et al. 2019).

Metazachlor [2-chloro-N-(2,6-dimethylphenyl)-N-(1H-pyrazol-1-ylmethyl)-acetamide] is a chloroacetamide herbicide (FAO 1999), with endocrine disruption as mode of action (Kralova et al. 2015). Thiacloprid {3-[(6-chloropyridin-3-yl)methyl]-1,3-thiazolidin-2-ylidene} cyanamide is a neurotoxic neonicotinoid insecticide (EPA 2003). Terbuthylazine [N^2^-tert-butyl-6-chloro-N4-ethyl-1,3,5-triazine-2,4-diamine] is a triazine herbicide (EFSA 2011) that can cause endocrine disruption (Ghisari et al. 2015). These pesticides are widely used in Central Europe (Hvezdova et al. 2018; Spitzer et al. 2020) and they have been reported in European surface waters (Table 1). Moreover, these pesticides have been found to negatively impact aquatic vertebrates and invertebrates at environmentally relevant concentrations (Guo et al. 2021; Gutierrez et al. 2019; Velisek and Stara 2018). Studies show that metazachlor induced changes in crayfish borrowing behavior and locomotor activity (Guo et al. 2021; Velisek et al. 2020). Zebrafish embryos exposed to thiacloprid exhibited altered avoidance and edge preference behaviors (Xie et al. 2022).

**Table 1:** Concentration of pesticides detected in European surface waters and concentrations used in this study

Pesticide class	Active substance	Range (mean) of reported concentrations (µg/L)	Concentration used in this study (µg/L)	Data sources
Neonicotinoids	Thiacloprid	0.02–12.0 (5.96)	6.0	Barmentlo et al. (2018), Sanchez-Bayo and Hyne (2014), and Suß et al. (2006)
Triazines	Terbuthylazine	0.02–13.0 (4.37)	4.0	Hermosin et al. (2013), Herrero-Hernandez et al. (2013), Herrero-Hernandez et al. (2017), and Lacorte et al. (1998)
Chloroacetanilide	Metazachlor	0.1–100.0 (25.8)	20.0	Kreuger (1998), Mohr et al. (2008), Ulrich et al. (2018), and Weber et al. (2018)

Among freshwater invertebrates, crayfish are considered keystone species because of their ecological and functional importance (Momot 1995). They can play a valuable role in monitoring environmental pollution through behavioral and physiological alterations and contaminant accumulation (Faria et al. 2010; Gago-Tinoco et al. 2014; Reisinger et al. 2021; Sohn et al. 2018). Non-native crayfishes are mostly used in toxicological studies due to the protected status of indigenous species (Buric et al. 2013; Velisek et al. 2020). Styrishave et al. (2007) found no differences in oxygen consumption and heart rate between native noble crayfish *Astacus astacus* and non-native signal crayfish *Pacifastacus leniusculus*. Such similarities can help to understand potential impacts on native crayfish populations, using the data from investigations with non-native species. Like many aquatic organisms, crayfish absorb chemicals from water through gills and the body surface in addition to ingesting pollutants along with prey (Katagi 2010). Crayfish are exposed to accumulated contaminants through contact with bottom sediments (Alcorlo et al. 2006) and are affected by pollutants, including pesticides, present in surface waters (Gago-Tinoco et al. 2014; Marcal et al. 2020; Sohn et al. 2018).

Pesticide concentrations in aquatic ecosystems increase with surface runoff (Liess et al. 1999) which is often episodic (Thurman et al. 1991), with concentrations varying depending on the time of application and precipitation events (Albanis et al. 1998). The majority of research into pesticide effects on crayfish focus on chronic exposure and show changes in crayfish antioxidant levels, histology, and behavior (Guo et al. 2021; Stara et al. 2020; Velisek et al. 2020). The response of crayfish to acute exposure to pesticides remains unclear. Since pulse exposure to pesticides has been reported to affect macroinvertebrates (Heckmann and Friberg 2005), it is important to know whether short-term pulses of agrochemicals adversely affect prime players in the freshwater environment, such as crayfish.

The objective of the present study was to quantify the acute response of the signal crayfish *P. leniusculus* to a brief pulse of metazachlor, terbuthylazine, or thiacloprid at environmentally relevant concentrations, as assessed by cardiac and locomotor activity. Crayfish have been known to exhibit alterations in cardiac and locomotor activity as responses to a wide variety of environmental stressors (Bini et al. 2015; Kuklina et al. 2014; Lozek et al. 2019; Velisek et al. 2020). In this study, changes in heart rate and distance moved were monitored to gain information of crayfish response to acute pesticide exposure.

## Materials and methods

### Chemicals

Metazachlor (MTZ), chemical purity 99.7%; terbuthylazine (TER), chemical purity 99.4%; and thiacloprid (TCL), chemical purity 99.9%, were purchased from Sigma-Aldrich Corporation (USA). Chemicals were dissolved in dechlorinated tap water to obtain 20 μg/L, 4 μg/L, and 6 μg/L for MTZ, TER, and TCL, respectively. Actual concentrations of chemicals in water during the experiments were within 96% of the nominal concentrations (Table 2). The analyses of pesticides in water were performed by the State Research Institute in Prague using methods described by Anastassiades et al. (2003) and Anastassiades et al. (2007).

**Table 2:** Concentrations of metazachlor (MTZ), terbuthylazine (TER), and thiacloprid (TCL) in exposure and control groups of signal crayfish *Pacifastacus leniusculus*

Group	Tank (*n*)	Nominal concentration (µg/L)	Concentration (µg/L) Mean ± SD	*t*	*p*-value
MTZ	6	20	19.3±1.5	-1.2	0.28
Control	6	˗	< 0.010	˗	˗
TER	6	4	3.9 ± 0.1	− 1.93	0.11
Control	6	˗	< 0.010	˗	˗
TCL	6	6	5.7 ± 0.3	− 2.18	0.08
Control	6	˗	< 0.010	˗	˗

*t*, *t*-score. *p* < 0.05. The limit of detection for the concentrations was 0.010 µg/L

### Test organisms

Thirty-six adult signal crayfish *Pacifastacus leniusculus* (1:1 male:female) were collected from Kresanovsky Brook (49°03′35.2″N, 13°45′33.8″E) near Sumava National Park, Czech Republic. Kresanovsky Brook is located in sub-mountain area and the majority of the watershed is forested with limited urban or agricultural land use. We used non-native crayfish species as indigenous species are endangered and manipulations with them are prohibited. Crayfish were transported to the laboratory and held in individual tanks in a recirculating aquarium system for pre-acclimatization. Both sexes of crayfish were used based on previous studies that found no significant differences between their reactions to stimuli or spatial behavior (Kuklina et al. 2018; Tierney and Andrews 2013). There were no risks associated with the escape of crayfish.

### Experimental protocol

The exposure concentrations were within the range reported in European surface waters (Table 1) although, because of the short exposure period, the experimental concentrations were higher than those used in long-term exposure studies (Englert et al. 2012; Guo et al. 2021).

The experiment was carried out in three phases, during which the crayfish were exposed to one of three pesticides (TCL, TER, or MTZ) or to dechlorinated tap water as control. The pesticides were each represented in a separate run. Each phase included 12 experimental crayfish: six exposed and six control specimens (3:3 male:female). Each of the three pesticide groups thus had its own control group. Heart rate was recorded using a non-invasive crayfish cardiac activity monitoring system (Pautsina et al. 2014). Briefly, this system consists of infrared (IR) sensors, a multichannel analog-to-digital converter (ADC) with USB interface, and a personal computer for data processing. The IR sensors were attached to the dorsal side of crayfish carapace above the heart with non-toxic epoxy glue. Wires that connect sensors and the ADC are flexible and allow crayfish to move freely. Heart rate was measured every second and then recorded as number of beats per minute (bpm).

To record movement, a Microsoft Kinect Sensor (Microsoft Corporation, Redmond, WA, USA) was placed under the tanks. Distance moved (cm) was measured every second and evaluated using a multiple-arena module in EthoVision XT 13.0 software (Noldus Information Technology, Wageningen, Netherlands).

Each crayfish with attached IR sensor was placed into separate non-recirculating 6-L tank (water temperature 20.3–21.5 °C, pH 7.6–7.8, dissolved oxygen 8.49–8.76 mg/L, 12:12-h light:dark cycle) for 10 days of acclimation and experimentation. The length of the tank wall was 30 cm and the width was 19 cm. The water depth in the tank was 11 cm. Twice weekly, chironomid larvae were provided and water was changed. Tanks were aerated to avoid disturbance to crayfish during pesticide application and to ensure rapid diffusion of the pesticide throughout the water. Plastic mesh was used as a substrate to provide crayfish with traction when moving. Three trials were conducted as follows: pesticide was administered to tanks simultaneously using individual peristaltic pumps. The compound is uniformly mixed in the tank during 30 s as authors tested prior to the experiment with colored liquid. Crayfish from the control group received dechlorinated tap water the same temperature as in experimental tanks. Crayfish heart rate and locomotor activity were recorded for 10 min before and 10 min after adding the pesticide. Therefore, crayfish were exposed to the pesticides for 10 min. Following the experiment, all crayfish were euthanized humanely by freezing at − 20 °C.

### Statistical analysis

All data were analyzed using Statistica v. 13 (StatSoft, Inc.). Prior to statistical analysis, the normality of the residuals was checked with Shapiro–Wilk’s test as the assumption for the analysis of variance (ANOVA), followed by Tukey’s test to compare differences between groups. The analysis was performed separately for each tested compound and followed parameters, comparing exposure group along with its dedicated control. The depended variables in each analysis were differences (after − before) in the heart rate and the distance moved. Categorical factors represented the treatment: control and exposure, respectively. Therefore, such an approach aimed to compare the changes of heart rate and locomotor activity in a response to the chemical exposure. To examine correlation of heart rate with locomotion after chemical exposure, simple linear regression was calculated to analyze increase of mean heart rate (after exposure relative to before) of each crayfish relative to the distance moved. All values are presented as mean ± standard deviation. Statistical significance was set at *p* < 0.05.

## Results

**Fig. 1:**
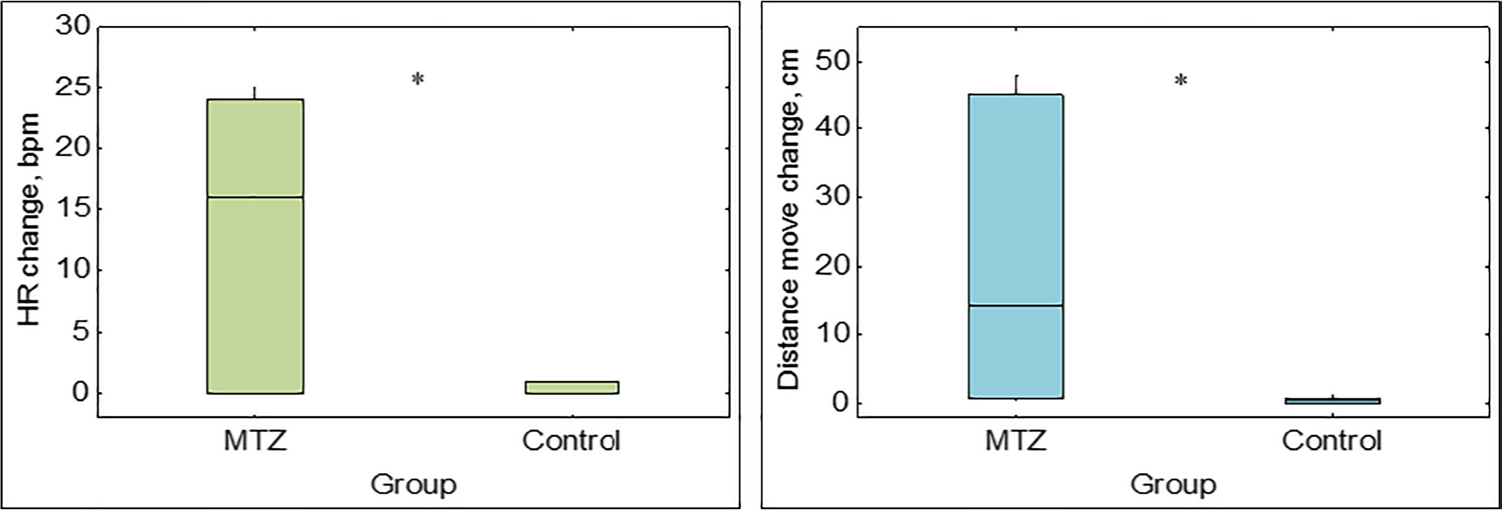
Changes in mean heart rate (HR) and distance moved of *Pacifastacus leniusculus* and controls before and after metazachlor (MTZ) exposure/water change; *bpm*, beats per minute. Significant differences (*p* < 0.05) are marked with asterisks (*)

**Fig. 2:**
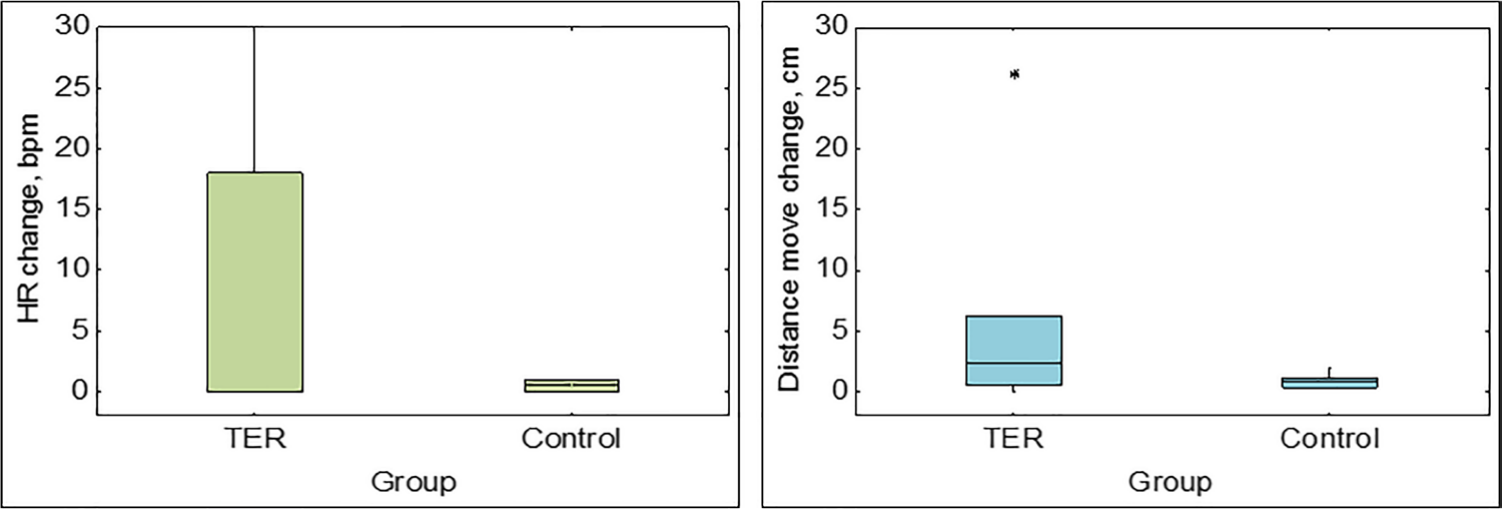
Changes in mean heart rate (HR) and distance moved of *Pacifastacus leniusculus* and controls before and after terbuthylazine (TER) exposure/water change; *bpm*, beats per minute

**Fig. 3:**
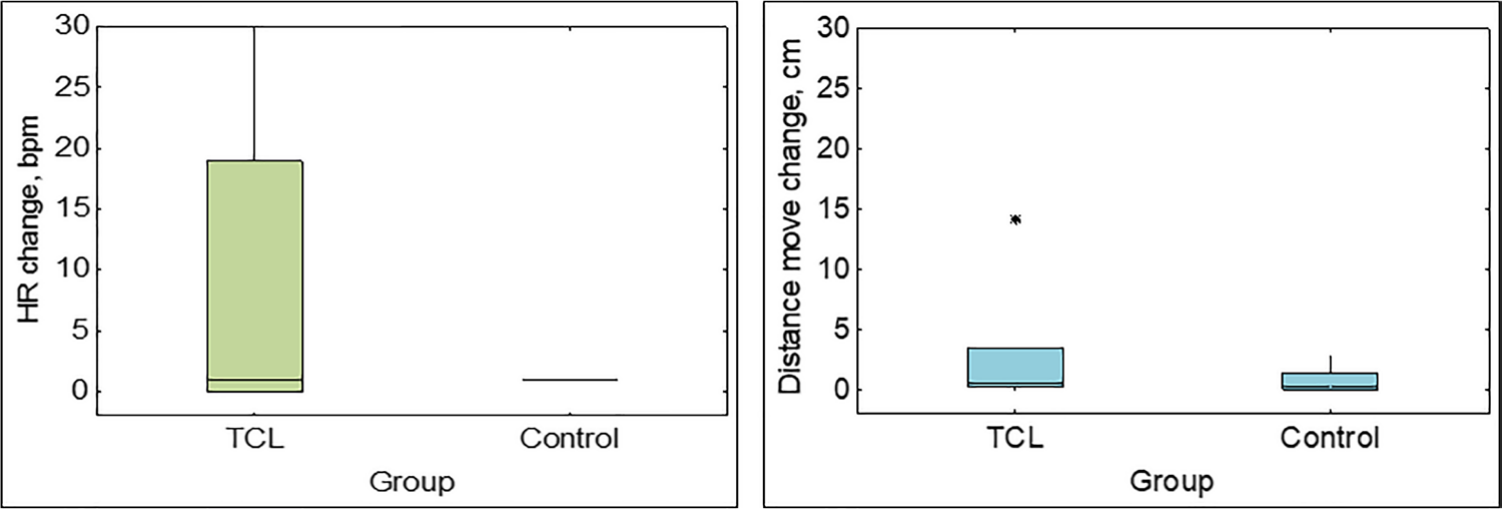
Changes in mean heart rate (HR) and distance moved of *Pacifastacus leniusculus* and controls before and after thiacloprid (TCL) exposure/water change; *bpm*, beats per minute

No significant differences were found in the biometrical parameters of the exposed and control groups of crayfish (Table S1). Changes in crayfish cardiac and locomotor activity after pesticide administration were observed in specimens of the group exposed to 20 μg/L of MTZ (Fig. 1). Significant changes in mean heart rate (*F*_1,10_ = 8.35, *p* = 0.016) and distance moved (*F*_1,10_ = 5.306, *p* = 0.044) after exposure compared to before were detected in treated crayfish. An increase in mean heart rate was detected at 118 ± 74 s post-exposure to MTZ. In the groups exposed to the concentrations of TER (4 μg/L) and TCL (6 μg/L) crayfish did not show a significant increase in mean heart rate (*F*_1,10_ = 1.973, *p* = 0.19; *F*_1,10_ = 2.019, *p* = 0.186) or distance moved (*F*_1,10_ = 1.726, *p* = 0.218; *F*_1,10_ = 1.051, *p* = 0.329) (Figs. 2 and 3). In these two groups, only 33% of specimens exhibited cardiac and locomotor response. There was no significant difference in mean heart rate or distance moved in all three control groups (*p* > 0.05) (Figs. 1, 2, and 3).

**Fig. 4:**
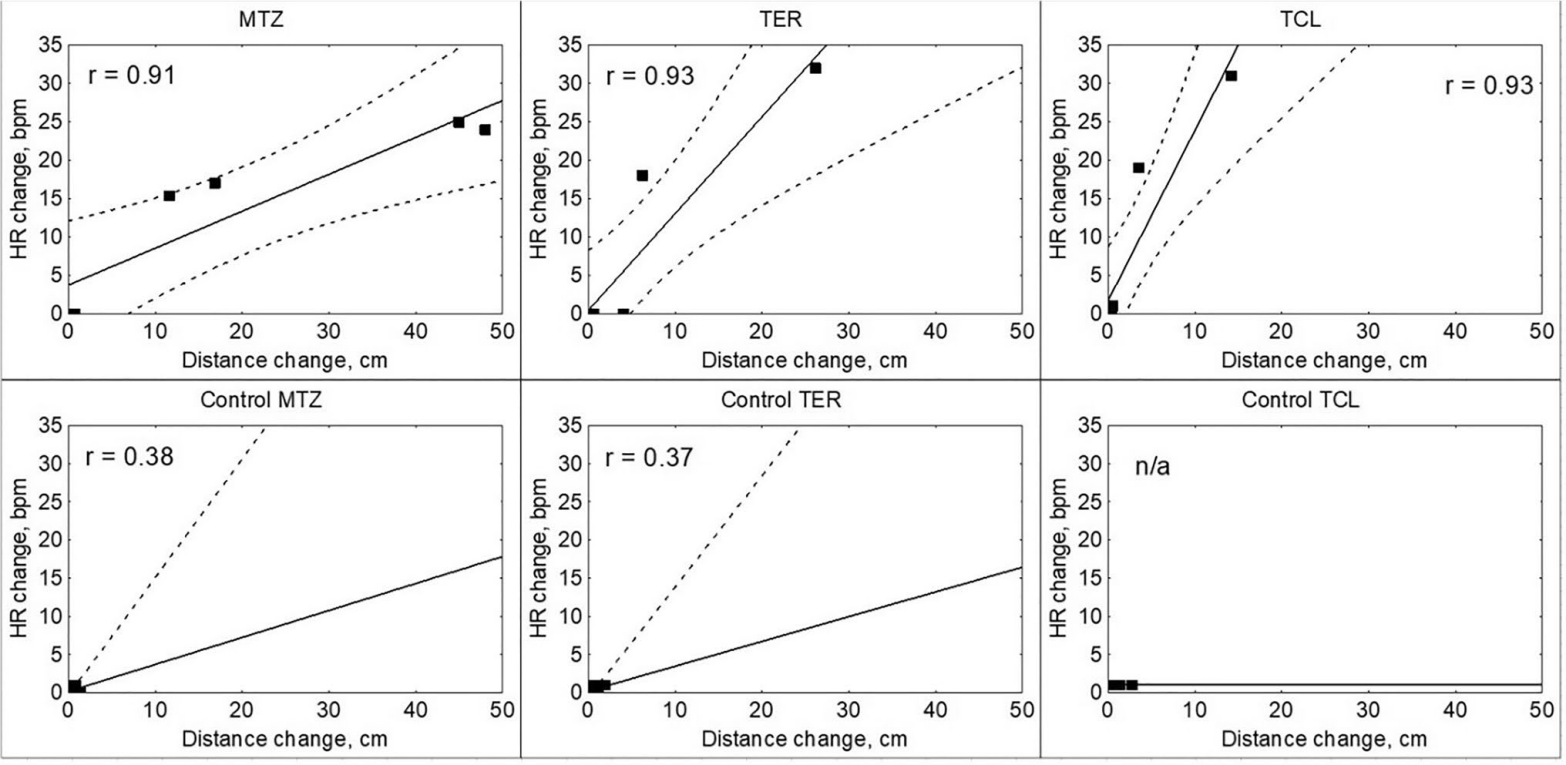
The relationship between mean heart rate (HR) and mean distance moved of *Pacifastacus leniusculus* exposed to metazachlor (MTZ), terbuthylazine (TER), and thiacloprid (TCL) and respective control groups. Pearson’s *r* = strength of the correlation between HR and distance moved

A linear regression model revealed a significant correlation between cardiac activity and distance moved in all exposure groups (Fig. 4), with the strongest response found in MTZ (*b* = 1.73), followed by TER (*b* = 0.68) and TCL (*b* = 0.39). Crayfish exposed to MTZ demonstrated four- and three-fold the movement response of those exposed to TCL and TER, respectively (Fig. 4). Changes in distance moved and heart rate showed correlation in all reacting crayfish.

## Discussion

As episodic spikes in chemical concentration are more typical of agricultural areas than a sustained release (Liess et al. 1999; Liess and Von der Ohe 2009), we quantified crayfish acute cardiac and locomotor responses to environmentally relevant pesticide concentrations. To our knowledge, this is the first study to report crayfish reaction to pulse exposure of pesticides in water.

A single exposure to chemicals at relatively high, but environmentally relevant, concentrations usually provokes detectable physiological or behavior changes (Beketov and Liess 2005, 2008a). With repeated exposure, macroinvertebrates exhibit a stronger response, possibly related to incomplete recovery from previous exposure (Berghahn et al. 2012; Mohr et al. 2012). Animals are often impacted by multiple simultaneous stressors, the effect of which depends on ambient physical and chemical parameters. The amphipod crustacean *Gammarus pulex* from contaminated streams was shown to be more sensitive to pesticide exposure than animals from an uncontaminated environment (Russo et al. 2018). Crayfish for the current experiment were obtained from a non-polluted ecosystem and acclimated to laboratory conditions for a prolonged time, so may have been less sensitive to the exposure.

With exposure to metazachlor, we detected significant changes in crayfish distance moved. An increase in locomotor activity can be the result of stimulatory effect of metazachlor. Previous studies have reported that pesticides can exhibit stimulatory effects in non-target organisms (Cutler et al. 2022; Guedes et al. 2009; Morse 1998) resulting in behavioral alterations in pesticide-exposed vertebrates and invertebrates (Deng et al. 2009; DuRant et al. 2007). Chemical irritation is often associated with stimulation of locomotor responses in aquatic organisms (Chen et al. 2014; Sharma 2019). The increased distance moved after exposure may also have represented active avoidance of the contaminated area. Velisek et al. (2019) documented an increase in distance moved in juvenile crayfish *Procambarus virginalis* exposed to the pesticide S-metolachlor. Buric et al. (2013) described attempts of crayfish *Faxonius limosus* and *Pacifastacus leniusculus* to escape exposure to the pesticide diazinon. Moreover, it has been reported that brief pesticide exposure can induce drift (Beketov and Liess 2008a) or increase drift density of a macroinvertebrate community (Heckmann and Friberg 2005). Sensitivity of ecosystem function and invertebrate population dynamics to environmental contaminants have been shown in several studies (Berenzen et al. 2005; Martin et al. 2011; Richmond et al. 2016, 2019). Drift of macroinvertebrates, driven by irritable or avoidance behavior, may lead to risks associated with predation, community structure alterations, decrease in abundance, and, consequently, affect the food chain.

Disorientation of crayfish in the presence of pesticides could be the result of temporary impairment of olfactory receptors (Cook and Moore 2008). Disruption of chemoreception can affect agonistic, feeding, and homing behavior, with juvenile crayfish potentially more sensitive to the impact of pesticides (Buric et al. 2013). The latter might partially explain the lack of reaction of some individuals in our study, since we examined adult crayfish. Metazachlor is an endocrine-disrupting agent that, among other effects, adversely impacts behavior and metabolism (Crisp et al. 1998). Increased cardiac and locomotor activity provides evidence of behavioral and metabolic disturbances in response to pesticide presence.

We did not observe significant changes in locomotor activity of crayfish exposed to terbuthylazine and thiacloprid at 4 and 6 μg/L, respectively, suggesting that, with such pesticide pulse, the majority of crayfish might not be stimulated to escape a contaminated area. This can lead to continuing exposure, as pesticide concentrations decrease over time (Ulrich et al. 2018). Concentrations as low as 0.5–1 μg/L of thiacloprid during a 96-h exposure were shown to adversely influence the predation activity of the aquatic invertebrate *Gammarus fossarum* (Englert et al. 2012). It is noteworthy that crayfish species may vary in level of sensitivity to a given substance. Buric et al. (2013) reported *P. leniusculus* to be less sensitive to diazinon treatment than was *F. limosus.* Species other than signal crayfish may exhibit greater physiological and behavior responses to terbuthylazine and thiacloprid at the tested concentrations. The low number of specimens reacting to thiacloprid might be connected with its mode of action. Like other neonicotinoids, thiacloprid stimulates nicotinic acetylcholine receptors in the central nervous system. While low activation of these receptors can manifest as nervous excitation, higher levels of thiacloprid can cause overexcitation and block the receptors, resulting in temporary paralysis (Yamamoto 1999), which may become more apparent with a longer exposure period.

In our experiment, changes in heart rate coincided with an increase in distance moved. This is in agreement with Kuklina et al. (2018), who demonstrated initiation of *Pontastacus leptodactylus* crayfish locomotion to coincide with heart rate increase as a reaction to chemical stimuli. With natural stimuli such as predator or conspecific crayfish odor, locomotion was delayed or was not manifested. Change in cardiac activity, in particular increased heart rate, is a typical stress response of crayfish to substances in water. This was demonstrated in studies of chemicals such as disinfectants, metals, and pharmaceuticals (Kuklina et al. 2014; Bini et al. 2015; Lozek et al. 2019). The cardiac response of *P. leniusculus* to tested pesticides confirms its potential to be used as a bioindicator of aquatic contamination by pesticides.

While we investigated the response of crayfish to an acute pulse of pesticide, some adverse effects might remain following the exposure. Evidence of impacts on survival and reproduction of *G. pulex* was detected for at least 2 weeks following a short pulse of the pyrethroid insecticide esfenvalerate at an environmentally relevant concentration (Cold and Forbes 2004). A single contamination event by thiacloprid can show effects on abundance and community structure of aquatic invertebrates after 7 days (Beketov et al. 2008) and, in community parameters, after 3 months (Liess and Beketov 2011). Delayed lethal and sublethal effects occurred in several freshwater crustacean species following a single thiacloprid exposure at a concentration of 5.47 µg/L (Beketov and Liess 2008b).

## Conclusions

The present work demonstrates that a short-term pulse of pesticide exposure can affect non-target organisms. Acute exposure to metazachlor at an environmentally relevant concentration can induce changes in crayfish heart rate and locomotor activity. With pulse exposure to terbuthylazine, and thiacloprid, the majority of animals might not respond to contaminants during short-term period. Owing to the prime role of crayfish in freshwater environment, the knowledge of how pesticides at environmentally relevant concentrations impact these crustaceans is of key importance. Spikes in pesticide concentrations are typical of aquatic environments, and further studies of the effect of a single short-term pesticide exposure on crayfish can reveal crucial information of the ecological consequences of such events.

## Supplementary Information

Below is the link to the electronic supplementary material.

Supplementary file1 (DOCX 14 KB)

## Data Availability

The dataset used and/or analyzed during the current study is available from the corresponding author on reasonable request.

## References

[CR1] Albanis TA, Hela DG, Sakellarides TM, Konstantinou IK (1998) Monitoring of pesticide residues and their metabolites in surface and underground waters of Imathia (N. Greece) by means of solid-phase extraction disks and gas chromatography. J Chromatogr A 823(1–2):59–71. 10.1016/S0021-9673(98)00304-5 10.1016/s0021-9673(98)00304-5 9818393

[CR2] Alcorlo P, Otero M, Crehuet M, Baltanas A, Montes C (2006). The use of the red swamp crayfish (*Procambarus clarkii*, Girard) as indicator of the bioavailability of heavy metals in environmental monitoring in the River Guadiamar (SW, Spain). Sci Total Environ.

[CR3] Anastassiades M, Mastovska K, Lehotay SJ (2003). Evaluation of analyte protectants to improve gas chromatographic analysis of pesticides. J Chromatogr A.

[CR4] Anastassiades M, Scherbaum E, Tasdelen B, Stajnbaher D (2007) Resent developments in QuEChERS methodology for pesticide multiresidue analysis. In: Ohkawa H, Miyagawa H, Lee PW (eds) Pesticide chemistry: crop protection, public health, environmental safety. Wiley-VCH Verlag GmbH & Co: KGaA, pp 439–458. 10.1002/9783527611249.ch46

[CR5] Barmentlo SH, Parmentier EM, de Snoo GR, Vijver MG (2018). Thiacloprid-induced toxicity influenced by nutrients: evidence from in situ bioassays in experimental ditches. Environ Toxicol Chem.

[CR6] Beketov MA, Liess M (2005). Acute contamination with esfenvalerate and food limitation: chronic effects on the mayfly Cloeon dipterum. Environ Toxicol Chem.

[CR7] Beketov MA, Liess M (2008). Potential of 11 pesticides to initiate downstream drift of stream macroinvertebrates. Arch Environ Contam Toxicol.

[CR8] Beketov MA, Liess M (2008). Acute and delayed effects of the neonicotinoid insecticide thiacloprid on seven freshwater arthropods. Environ Toxicol Chem.

[CR9] Beketov MA, Schafer RB, Marwitz A, Paschke A, Liess M (2008). Long-term stream invertebrate community alterations induced by the insecticide thiacloprid: effect concentrations and recovery dynamics. Sci Total Environ.

[CR10] Benbrook CM (2016). Trends in glyphosate herbicide use in the United States and globally. Environ Sci Eur.

[CR11] Berenzen N, Kumke T, Schulz HK, Schulz R (2005). Macroinvertebrate community structure in agricultural streams: impact of runoff-related pesticide contamination. Ecotoxicol Environ Saf.

[CR12] Berghahn R, Mohr S, Hubner V, Schmiediche R, Schmiedling I, Svetich-Will E, Schmidt R (2012). Effects of repeated insecticide pulses on macroinvertebrate drift in indoor stream mesocosms. Aquat Toxicol.

[CR13] Bini G, Santini G, Chelazzi G (2015). Pre-exposure to cadmium or zinc alters the heart rate response of the crayfish *Procambarus clarkii* towards copper. Bull Environ Contam Toxicol.

[CR14] Bunzel K, Schafer RB, Thran D, Kattwinkel M (2015). Pesticide runoff from energy crops: a threat to aquatic invertebrates?. Sci Total Environ.

[CR15] Buric M, Kouba A, Machova J, Mahovska I, Kozak P (2013). Toxicity of the organophosphate pesticide diazinon to crayfish of differing age. Int J Environ Sci Technol.

[CR16] Chen TH, Lin CC, Meng PJ (2014). Zinc oxide nanoparticles alter hatching and larval locomotor activity in zebrafish (*Danio rerio*). J Hazard Mater.

[CR17] Cold A, Forbes VE (2004). Consequences of a short pulse of pesticide exposure for survival and reproduction of *Gammarus pulex*. Aquat Toxicol.

[CR18] Cook ME, Moore PA (2008). The effects of the herbicide metolachlor on agonistic behavior in the crayfish Orconectes rusticus. Arch Environ Contam Toxicol.

[CR19] Crisp TM, Clegg ED, Cooper RL, Wood WP, Anderson DG, Baetcke KP, Hoffmann JL, Morrow MS, Rodier DJ, Schaeffer JE, Touart LW, Zeeman MG, Patel YM (1998). Environmental endocrine disruption: an effects assessment and analysis. Environ Health Perspect.

[CR20] Cutler GC, Amichot M, Benelli G, Guedes RNC, Qu Y, Rix RR, Ullah F, Desneux N (2022) Hormesis and insects: effects and interactions in agroecosystems. Sci Total Environ 825:153899. 10.1016/j.scitotenv.2022.153899 10.1016/j.scitotenv.2022.153899 35181361

[CR21] De Geronimo E, Aparicio VC, Barbaro S, Portocarrero R, Jaime S, Costa JL (2014). Presence of pesticides in surface water from four sub-basins in Argentina. Chemosphere.

[CR22] Deng L, Dai J, Xu M (2009). Effects of methamidophos on the predating behavior of *Hylyphantes graminicola* (Sundevall) (Araneae: Linyphiidae). Environ Toxicol Chem.

[CR23] Dobrovolski R, Diniz-Filho JAF, Loyola RD, Junior PDM (2001). Agricultural expansion and the fate of global conservation priorities. Biodivers Conserv.

[CR24] DuRant SE, Hopkins WA, Talent LG (2007). Impaired terrestrial and arboreal locomotor performance in the western fence lizard (*Sceloporus occidentalis*) after exposure to an AChE-inhibiting pesticide. Environ Pollut.

[CR25] EFSA (European Food Safety Authority) (2011) Conclusion on the peer review of the pesticide risk assessment of the active substance terbuthylazine. European Food Safety Authority Journal 9(1):196910.2903/j.efsa.2011.1969 10.2903/j.efsa.2011.2087 PMC13177329 42148408

[CR26] Englert D, Bundschuh M, Schulz R (2012). Thiacloprid affects trophic interaction between gammarids and mayflies. Environ Pollut.

[CR27] EPA (Environmental Protection Agency) (2003) Fact sheet for thiacloprid. United States. Office of Prevention and Toxic Substances (7501C)

[CR28] FAO (1999) FAO (Food and Agriculture Organization) specifications and evaluations for plant protection products. Metazachlor, 17

[CR29] Faria M, Huertas D, Soto DX, Grimalt JO, Catalan J, Riva MC, Barata C (2010). Contaminant accumulation and multi-biomarker responses in field collected zebra mussels (*Dreissena polymorpha*) and crayfish (*Procambarus clarkii*), to evaluate toxicological effects of industrial hazardous dumps in the Ebro river (NE Spain). Chemosphere.

[CR30] Gago-Tinoco A, Gonzalez-Dominguez R, Garcia-Barrera T, Blasco-Moreno J, Bebianno MJ, Gomez-Ariza JL (2014). Metabolic signatures associated with environmental pollution by metals in Donana National Park using *P. clarkii* as bioindicator. Environ Sci Pollut Res.

[CR31] Gao J, Liu L, Liu X, Lu J, Zhou H, Huang S, Wang Z, Spear PA (2008). Occurrence and distribution of organochlorine pesticides — lindane, *p*, p′-DDT, and heptachlor epoxide — in surface water of China. Environ Int.

[CR32] Ghisari M, Long M, Tabbo A, Bonefeld-Jorgensen EC (2015). Effects of currently used pesticides and their mixtures on the function of thyroid hormone and aryl hydrocarbon receptor in cell culture. Toxicol Appl Pharmacol.

[CR33] Guedes RNC, Magalhaes LC, Cosme LV (2009). Stimulatory sublethal response of a generalist predator to permethrin: hormesis, hormoligosis, or homeostatic regulation?. J Econ Entomol.

[CR34] Guo W, Weiperth A, Hossain MS, Kubec J, Grabicova K, Lozek F, Vesely L, Blaha M, Buric M, Kouba A, Velisek J (2021) The effects of the herbicides terbuthylazine and metazachlor at environmental concentration on the burrowing behaviour of red swamp crayfish. Chemosphere 270:128656. 10.1016/j.chemosphere.2020.128656 10.1016/j.chemosphere.2020.128656 33172666

[CR35] Gutierrez IB, Mesquita AFC, Nunes C, Coimbra MA, Goncalves FJM, Marques JC, Goncalves AMM (2019). Impacts of S-metolachlor and terbuthylazine in fatty acid and carbohydrate composition of the benthic clam *Scrobicularia plana*. Ecotoxicol Environ Saf.

[CR36] Heckmann LH, Friberg N (2005). Macroinvertebrate community response to pulse exposure with the insecticide lambda-cyhalothrin using in-stream mesocosms. Environ Toxicol Chem.

[CR37] Hermosin MC, Calderon MJ, Real M, Cornejo J (2013). Impact of herbicides used in olive groves on waters of the Guadalquivir River basin (southern Spain). Agric Ecosyst Environ.

[CR38] Herrero-Hernandez E, Andrades MS, Alvarez-Martin A, Pose-Juan E, Rodriguez-Cruz MS, Sanchez-Martin MJ (2013). Occurrence of pesticides and some of their degradation products in waters in Spanish wine region. J Hydrol.

[CR39] Herrero-Hernandez E, Rodriguez-Cruz MS, Pose-Juan E, Sanchez-Gonzalez S, Andrades MS, Sanchez-Martin MJ (2017). Seasonal distribution of herbicide and insecticide residues in the water resources of the vineyard region of La Rioja (Spain). Sci Total Environ.

[CR40] Herrero-Hernandez E, Simon-Egea AB, Sanchez-Martin MJ, Rodriguez-Cruz MS, Andrades MS (2020) Monitoring and environmental risk assessment of pesticide residues and some of their degradation products in natural waters of the Spanish vineyard region included in the Denomination of Origin Jumilla. Environ Pollut 264:144666. 10.1016/j.envpol.2020.114666 10.1016/j.envpol.2020.114666 32380396

[CR41] Hvezdova M, Kosubova P, Kosikova M, Scherr KE, Simek Z, Brodsky L, Sudoma M, Skulcova L, Sanka M, Svobodova M, Krkoskova L, Vasickova J, Neuwirthova N, Bielska L, Hofman J (2018). Currently and recently used pesticides in Central European arable soils. Sci Total Environ.

[CR42] Jergentz S, Mugni H, Bonetto C, Schulz R (2005). Assessment of insecticide contamination in runoff and stream water of small agricultural streams in the main soybean area of Argentina. Chemosphere.

[CR43] Katagi T (2010). Bioconcentration, bioaccumulation, and metabolism of pesticides in aquatic organisms. Rev Environ Contam Toxicol.

[CR44] Kralova M, Levchuk I, Kasparek V, Sillanpaa M, Cihlar J (2015). Influence of synthesis conditions on physical properties of lanthanide-doped titania for photocatalytic decomposition of metazachlor. Chinese J Catal.

[CR45] Kreuger J (1998). Pesticides in stream water within an agricultural catchment in southern Sweden, 1990–1996. Sci Total Environ.

[CR46] Kuklina I, Lozek F, Cisar P, Kouba A, Kozak P (2018). Crayfish can distinguish between natural and chemical stimuli as assessed by cardiac and locomotor reactions. Environ Sci Pollut Res.

[CR47] Kuklina I, Sladkova S, Kouba A, Kholodkevich S, Kozak P (2014). Investigation of chloramine-T impact on crayfish *Astacus leptodactylus* (Esch., 1823) cardiac activity. Environ Sci Pollut Res.

[CR48] Lacorte S, Vreuls JJ, Salau JS, Ventura F, Barcelo D (1998). Monitoring of pesticides in river water using fully automated on-line solid-phase extraction and liquid chromatography with diode array detection with a novel filtration device. J Chromatogr A.

[CR49] Liess M, Beketov M (2011). Traits and stress: keys to identify community effects of low levels of toxicants in test systems. Ecotoxicology.

[CR50] Liess M, Schulz R, Liess MHD, Rother B, Kreuzig R (1999). Determination of insecticide contamination in agricultural headwater streams. Water Res.

[CR51] Liess M, Von der Ohe PC (2009). Analyzing effects of pesticides on invertebrate communities in streams. Environ Toxicol Chem.

[CR52] Lozek F, Kuklina I, Grabicova K, Kubec J, Buric M, Grabic R, Randak T, Cisar P, Kozak P (2019) Behaviour and cardiac response to stress in signal crayfish exposed to environmental concentrations of tramadol. Aquatic Toxicology 213:105217. 10.1016/j.aquatox.2019.05.019 10.1016/j.aquatox.2019.05.019 31200331

[CR53] Marcal R, Pacheco M, Guilherme S (2020) DNA of crayfish spermatozoa as a target of waterborne pesticides — an ex vivo approach as a tool to short-term spermiotoxicity screening. J Hazard Mater 400:123300. 10.1016/j.jhazmat.2020.123300 10.1016/j.jhazmat.2020.123300 32947705

[CR54] Martin S, Bertaux A, Le Ber F, Maillard E, Imfeld G (2011). Seasonal changes of macroinvertebrate communities in a stormwater wetland collecting pesticide runoff from a vineyard catchment (Alsace, France). Arch Environ Contam Toxicol.

[CR55] Matin MA, Malek MA, Amin MR, Rahman S, Khatoon J, Rahman M, Aminuddin M, Mian AJ (1998). Organochlorine insecticide residues in surface and underground water from different regions of Bangladesh. Agric Ecosyst Environ.

[CR56] Mohr S, Berghahn R, Schmiediche R, Hubner V, Loth S, Feibicke M, Mailahn W, Wogram J (2012). Macroinvertebrate community response to repeated short-term pulses of the insecticide imidacloprid. Aquat Toxicol.

[CR57] Mohr S, Feibicke M, Berghahn R, Schmiediche R, Schmidt R (2008). Response of plankton communities in freshwater pond and stream mesocosms to the herbicide metazachlor. Environ Pollut.

[CR58] Momot WD (1995). Redefining role of crayfish in aquatic ecosystem. Reviews Fish Sci.

[CR59] Morse JG (1998). Agricultural implications of pesticide-induced hormesis of insects and mites. Hum Exp Toxicol.

[CR60] Oerke EC (2006). Crop losses to pests. J Agric Sci.

[CR61] Palma P, Kock-Schulmeyer M, Alvarenga P, Ledo L, Barbosa IR, de Alda ML, Barcelo D (2014). Risk assessment of pesticides detected in surface water of the Alqueva reservoir (Guadiana basin, southern of Portugal). Sci Total Environ.

[CR62] Papadakis EN, Tsaboula A, Vryzas Z, Kotopoulou A, Kintzikoglou K, Papadopoulou-Mourkidou E (2018). Pesticides in the rivers and streams of two river basins in northern Greece. Sci Total Environ.

[CR63] Pautsina A, Kuklina I, Stys D, Cisar P (2014). Noninvasive crayfish cardiac activity monitoring system. Limnol Oceanogr Methods.

[CR64] Reisinger AJ, Reisinger LS, Richmond EK, Rosi EJ (2021) Exposure to a common antidepressant alters crayfish behavior and has potential subsequent ecosystem impacts. Ecosphere 12(6):e03529. 10.1002/ecs2.3527

[CR65] Richmond EK, Rosi EJ, Reisinger AJ, Hanrahan BR, Thompson RM, Grace MR (2019). Influences of the antidepressant fluoxetine on stream ecosystem function and aquatic insect emergence at environmentally realistic concentrations. J Freshw Ecol.

[CR66] Richmond EK, Rosi-Marshall EJ, Lee SS, Thompson RM, Grace MR (2016). Antidepressant in stream ecosystems: influence of selective serotonin reuptake inhibitors (SSRIs) on algal production and insect emergence. Freshw Sci.

[CR67] Rosi-Marshall EJ, Tank JL, Royer TV, Whiles MR, Evans-White M, Chambers C, Griffiths NA, Pokelsek J, Stephen ML (2007). Toxins in transgenic crop byproducts may affect headwater stream ecosystems. Proc Natl Acad Sci USA.

[CR68] Russo R, Becker JM, Liess M (2018). Sequential exposure to low levels of pesticides and temperature stress increase toxicological sensitivity of crustaceans. Sci Total Environ.

[CR69] Sanchez-Bayo F, Hyne RV (2014). Detection and analysis of neonicotinoids in river waters — development of a passive sampler for three commonly used insecticides. Chemosphere.

[CR70] Sharma M (2019) Behavioural responses in effect to chemical stress in fish: a review. Int J Fish Aquat Stud 7)1):1–5

[CR71] Sohn L, Brodie RJ, Couldwell G, Demmons E, Sturve J (2018). Exposure to a nicotinoid pesticide reduces defensive behaviors in a non-target organism, the rusty crayfish *Orconectes rusticus*. Ecotoxicology.

[CR72] Spitzer T, Bilovsky J, Matusinsky P (2020) Changes in resistance development in pollen beetle (*Brassicogethes aeneus* F.) to lambda-cyhalothrin, etofenprox, chlorpyrifos-ethyl, and thiacloprid in the Czech Republic during 2013–2017. Crop Prot 135:105224. 10.1016/j.cropro.2020.105224

[CR73] Stara A, Kubec J, Zuskova E, Buric M, Faggio C, Kouba A, Velisek J (2019). Effects of S-metolachlor and its degradation product metolachlor OA on marbled crayfish (*Procambarus virginalis*). Chemosphere.

[CR74] Stara A, Zuskova E, Vesely L, Kouba A, Velisek J (2020) Single and combined effects of thiacloprid concentration, exposure duration, and water temperature on marbled crayfish *Procambarus virginalis*. Chemosphere 17:128463. 10.1016/j.chemosphere.2020.128463 10.1016/j.chemosphere.2020.128463 34756343

[CR75] Styrishave B, Bojsen BH, Witthofft H, Andersen O (2007). Diurnal variations in physiology and behaviour of the noble crayfish *Astacus astacus* and the signal crayfish *Pacifastacus leniusculus*. Mar Freshw Behav Physiol.

[CR76] Suß A, Bischoff G, Mueller ACW, Buhr L (2006). Chemisch-biologisches Monitoring zu Pflanzenschutzmittelbelastungen und Lebensgemeinschaften in Gräben des Alten Landes. Nachrichtenblatt Des Deutschen Pflanzenschutzdienstes.

[CR77] Thurman EM, Goolsby DA, Meyer MT, Kolpin DW (1991). Herbicides in surface waters of the Midwestern United States: the effect of spring flush. Environ Sci Technol.

[CR78] Tierney AJ, Andrews K (2013). Spatial behavior in male and female crayfish (*Orconectes rusticus*): learning strategies and memory duration. Anim Cogn.

[CR79] Ulrich U, Hormann G, Unger M, Pfannerstill M, Steinmann F, Fohrer N (2018). Lentic small water bodies: variability of pesticide transport and transformation patterns. Sci Total Environ.

[CR80] Velisek J, Stara A (2018). Effect of thiacloprid on early life stages of common carp (*Cyprinus carpio*). Chemosphere.

[CR81] Velisek J, Stara A, Kubec J, Zuskova E, Buric M, Kouba A (2020). Effects of metazachlor and its major metabolite metazachlor OA on early life stages of marbled crayfish. Sci Rep.

[CR82] Velisek J, Stara A, Zuskova E, Kubec J, Buric M, Kouba A (2019). Effects of S-metolachlor on early life stages of marbled crayfish. Pestic Biochem Physiol.

[CR83] Wan Y, Tran TM, Nguyen VT, Wang A, Wang J, Kannan K (2021) Neonicotinoids, fipronil, chlorpyrifos, carbendazim, chlorotriazines, chlorophenoxy herbicides, bentazon, and selected pesticide transformation products in surface water and drinking water from northern Vietnam. Sci Total Environ 750:141507. 10.1016/j.scitotenv.2020.141507 10.1016/j.scitotenv.2020.141507 32841807

[CR84] Weber G, Christmann N, Thiery AC, Martens D, Kubiniok J (2018). Pesticides in agricultural headwater streams in southwestern Germany and effects on macroinvertebrate populations. Sci Total Environ.

[CR85] Xie Z, Guanghua L, Yeting Y (2022). Early-stage high-concentration thiacloprid exposure induced persistent behavioral alterations in zebrafish. Int J Environ Res Public Health.

[CR86] Yamamoto I (1999) Nicotine to nicotinoids: 1962 to 1997. In: Yamamoto I, Casida J (eds) Nicotinoid Insecticides and the Nicotinic Acetylcholine Receptor. Springer-Verlag, Tokyo, Japan, pp 3–27. 10.1007/978-4-431-67933-2

