# Supplementary material for: Responses of signal crayfish Pacifastacus leniusculus to single short-term pulse exposure of pesticides at environmentally relevant concentrations
Source: Environ Sci Pollut Res Int. 2023 Feb 23;30(18):51740–8. doi: 10.1007/s11356-023-25908-7 (PMC10119208; doi:10.1007/s11356-023-25908-7)
Supplement: Supplementary file 1 — Supplementary file1 (DOCX 14 KB) [file 11356_2023_25908_MOESM1_ESM.docx]

**Supplementary Information**

Responses of signal crayfish *Pacifastacus leniusculus* tosingle short-term pulse exposure of pesticides at environmentally relevant concentrations

Environmental Science and Pollution Research

Viktoriia Malinovska*, Iryna Kuklina, Filip Lozek, Josef Velisek, Pavel Kozak

*University of South Bohemia in Ceske Budejovice, Czech Republic*

*Corresponding author.

*E-mail address*: [vmalinovska@frov.jcu.cz](mailto:vmalinovska@frov.jcu.cz) (V. Malinovska)

**Table S1** Comparison of carapace length (CL) and total length (TL) of signal crayfish *Pacifastacus leniusculus* used in the study. MTZ (metazachlor), TER (terbuthylazine), TCL (thiacloprid). Data are presented as mean ± standard deviation; p < 0.05

| Parameters | MTZ | Control | ANOVA^1^  P-value,  F-statistics | TER | Control | ANOVA  P-value, F-statistics | TCL | Control | ANOVA  P-value,  F-statistics |
| --- | --- | --- | --- | --- | --- | --- | --- | --- | --- |
|  |  |  |  |  |  |  |  |  |  |
|  |  |  |  |  |  |  |  |  |  |
| CL (mm) | 42.8±1.4 | 42.3±1.7 | F(1, 13)=0.301, p=0.592 | 41.7±1.7 | 41.6±1.3 | F(1, 13)=0.013, p=0.910 | 42.7±1.6 | 42.7±2.0 | F(1, 13)=0.005, p=0.945 |
|  |  |  |  |  |  |  |  |  |  |
| TL (mm) | 87.5±1.8 | 86.1±2.5 | F(1, 13)=1.610, p=0.227 | 85.0±1.8 | 85.3±1.9 | F(1, 13)=0.067, p=0.800 | 86.2±3.2 | 87.1±3.9 | F(1, 13)=0.216, p=0.650 |

*1 – one-way ANOVA*
